# Responses of soil respiration to soil management changes in an agropastoral ecotone in Inner Mongolia, China

**DOI:** 10.1002/ece3.3659

**Published:** 2017-11-26

**Authors:** Haili Xue, Haiping Tang

**Affiliations:** ^1^ State Key Laboratory of Earth Surface Processes and Resource Ecology Faculty of Geographical Science Beijing Normal University Beijing China

**Keywords:** cropland, grazing grassland, restoration grassland, soil temperature, soil water content

## Abstract

Studying the responses of soil respiration (*R*_s_) to soil management changes is critical for enhancing our understanding of the global carbon cycle and has practical implications for grassland management. Therefore, the objectives of this study were (1) quantify daily and seasonal patterns of *R*_s_, (2) evaluate the influence of abiotic factors on *R*_s_, and (3) detect the effects of soil management changes on *R*_s_. We hypothesized that (1) most of daily and seasonal variation in *R*_s_ could be explained by soil temperature (*T*_s_) and soil water content (*S*_w_), (2) soil management changes could significantly affect *R*_s_, and (3) soil management changes affected *R*_s_ via the significant change in abiotic and biotic factors. In *situ R*_s_ values were monitored in an agropastoral ecotone in Inner Mongolia, China, during the growing seasons in 2009 (August to October) and 2010 (May to October). The soil management changes sequences included free grazing grassland (FG), cropland (CL), grazing enclosure grassland (GE), and abandoned cultivated grassland (AC). During the growing season in 2010, cumulative *R*_s_ for FG, CL, GE, and AC averaged 265.97, 344.74, 236.70, and 226.42 gC m^−2^ year^−1^, respectively. The *T*_s_ and *S*_w_ significantly influenced *R*_s_ and explained 66%–86% of the variability in daily *R*_s_. Monthly mean temperature and precipitation explained 78%–96% of the variability in monthly *R*_s_. The results clearly showed that *R*_s_ was increased by 29% with the conversion of FG to CL and decreased by 35% and 11% with the conversion of CL to AC and FG to GE. The factors impacting the change in *R*_s_ under different soil management changes sequences varied. Our results confirm the tested hypotheses. The increase in *Q*_*1*0_ and litter biomass induced by conversion of FG to GE could lead to increased *R*_s_ if the climate warming. We suggest that after proper natural restoration period, grasslands should be utilized properly to decrease *R*_s_.

## 1 INTRODUCTION

Soil respiration (*R*_s_) is a crucial process in the global carbon cycle (Bahn, Janssens, Reichstein, Smith, & Trumbore, 2010). Minor changes in *R*_s_ have the potential to significantly affect atmospheric CO_2_ concentrations (IPCC, 2007). Large‐scale soil management changes have been affecting *R*_s_, with considerable impacts on the terrestrial ecosystem carbon cycle. It has been estimated that global net flux due to land use change during the period of 1,850–2,000 is 148.6 Pg C (Kaul, Dadhwal, & Mohren, 2009); however, the mechanisms of this effect remain subject of debate (Nazaries et al., 2015). In recent years, considerable efforts have been made to understand the effects of soil management changes, that is, conversion of cropland to woodland or forest (Kellman, Beltrami, & Risk, 2007; Saurette, Chang, & Thomas, 2007; Zhang et al., 2015), forest to cropland, or grassland (Sheng et al., 2010). However, few studies have focused on the conversion of grazed grassland to cropland, and not many studies focus on conversion of cropland to grassland in degraded ecosystems (Shi, Yan, Zhang, Guan, & Du, 2014; Zhang et al., 2015).

Soil management changes can potentially alter soil temperature (*T*_s_) and soil water content (*S*_w_) (Chen et al., 2016; Rong, Ma, Johnson, & Yuan, 2015; Wang, Gong, et al., 2015), which are the main abiotic factors affecting *R*_s_ (Fang & Moncrieff, 2001; Gomez‐Casanovas, Matamala, Cook, & Gonzalez‐Meler, 2012), and these two factors affect the productivity and the decomposition rate of soil organic matter (Han et al., 2007). Temperature sensitivity (*Q*_10_) describes the relationship between *R*_s_ and temperature and can therefore also be changed with soil management changes (Gong et al., 2014; Rong et al., 2015). Furthermore, soil management changes can impact biotic factors, such as net primary production, belowground biomass (BGB), soil organic carbon (SOC) (Deng, Liu, & Shangguan, 2014; Frank, Liebig, & Tanaka, 2006; Sheng et al., 2010; Zhang et al., 2015), all of which greatly affect *R*_s_. However, the effects of soil management changes from cropland to grassland on *R*_s_ have not been consistent among studies, some studies indicate that it increases *R*_s_ (Frank et al., 2006; Wang, Liu, et al., 2015), while other studies show that it reduces *R*_s_ (Iqbal et al., 2008; Zhang et al., 2015). Moreover, the effects of grazing on *R*_s_ also have no consistent conclusion (Rong et al., 2015). Therefore, additional studies are needed to clarify the effects of the soil management changes on *R*_s_.

The northern agropastoral ecotone of China, which is a transition zone between agricultural and pastoral regions and encompasses various ecosystems, occupies an area of 8 × 10^5^ km^2^. Soil management changes from grassland to cropland or from cropland to grassland are frequently occurring in this region, making it the most sensitive eco‐environmental area in China (Zhou et al., 2007). In this area, the typical soil management changes sequences have occurred, including the conversion of free grazing grassland (FG) to cropland (CL), both of which are under human disturbance, and the conversion of FG and CL to restoration grassland—grazing enclosure grassland (GE) and abandoned cultivated grassland (AC). This variety of different soil management changes provides a unique opportunity to study the response of *R*_s_ to soil management changes. Previous studies of *R*_s_ in the temperate grassland in China primarily focused on the influence of grassland management practices on *R*_s_ (Li & Sun, 2011; Lu, Liao, & Liao, 2016; Su, Li, Cui, & Zhao, 2005), further studies are needed to study the impacts of conversions from FG to CL and GE, CL to AC on *R*_s_, biotic (aboveground biomass (AGB), BGB, SOC, etc.), and abiotic factors (*T*_s_, *S*_w_).

In this study, we measured *R*_s_, AGB, BGB, SOC, and soil microclimate in degraded areas of the agropastoral ecotone (soil management types: FG, CL, GE, and AC) in Inner Mongolia from 2009 to 2010. The objectives of our study were to (1) quantify daily and seasonal patterns of *R*_s_ in four soil management types, (2) evaluate the influence of abiotic (*T*_s_, *S*_w_) factors on *R*_s_ in these soil management types, and (3) detect the effects of soil management changes on *R*_s_.

We hypothesized that (1) most of daily and seasonal variation in *R*_s_ could be explained by *T*_s_ and *S*_w_, (2) soil management changes could significantly affect *R*_s_ and (3) soil management changes affected *R*_s_ via the significant change in abiotic and biotic factors.

## 2 MATERIALS AND METHODS

### 2.1 Site descriptions

The study was conducted in Duolun County in Inner Mongolia (15.83°–116.92°N, 41.77°–42.65°E, 1,150–1,800 m asl), located on the south edge of the Inner Mongolia Plateau, which belongs to a typical agropastoral ecotone in Northern China with a semi‐arid monsoon climate. The long‐term (1952–2009) mean annual temperature is 2.3°C, and the average temperature for July and January are 19.0°C and −17.5°C, respectively. Annual evaporation is 1,748 mm, and annual precipitation is 382 mm and accounts for 70% of the year from June to August. The soil has been classified as chestnut soil in the Chinese Soil Classification Standard (State Soil Survey Service of China, 1998) or as haplic calcisols by the Food and Agricultural Organization (FAO) of the United Nations.

Four adjacent experimental sites were established in the study area: free grazing grassland (FG), cropland (CL), grazing enclosure grassland (GE), and abandoned cultivated grassland (AC). The sites were flat terrain and located 150–800 m apart. The 11‐ha FG site (42.04°N, 116.29°E) has been consistently grazed at a stocking rate of 9 sheep/ha during the growing season, and the dominated species are *Stipa krylovli* Roshev and *Leymus chinensis* (Trin.) Tzvel. The 20‐ha CL site (42.04°N, 116.28°E) converted from FG and ploughed in 2008 for the grown of *Triticum aestivum* L. or *Fagopyrum sagittatum* Gilib each year, which were harvested by the end of September; the soil was ploughed about 20 cm and manure was applied at 80 kg/ha about 3 weeks before sowing, and no irrigation was applied in the site. GE and AC were enclosed grassland for the natural restoration of FG and CL. The 10‐ha GE site (42.04°N, 116.29°E) converted from FG, and it had not been grazed since 2000 and was preserved as a natural grassland dominated by *Stipa krylovii* Roshev, *Leymus chinensis* (Trin.) Tzvel. and *Artemisia frigida* Willd. The 13‐ha AC site (42.04°N, 116.28°E) converted from cropland (which had been converted from FG) in 2000, and *Agropyron cristatum* (Linn.) Gaertn. was planted at the enclosed first year.

### 2.2 Measurement of *R*_s_, *T*_s_, and *S*_w_

In each site, five 2 × 2 m sample plots were randomly selected with the constraint that the plots were located at least 10 m from the edge of the site to avoid edge effects. One day before the measurements, PVC collars were inserted (20 cm in diameter by 5 cm in height) 2 cm into the soil at each plot. To exclude respiration from aboveground vegetation, all visible living plants were removed from the collars before measurements. The measurements were taken eight times per day at 3‐hr intervals from 6:00 a.m. to 03:00 p.m., and instantaneous *R*_s_ in each plot was measured three times with an LI‐8100 Automated Soil CO_2_ Flux System (LI‐COR Environmental, Lincoln, NE, USA) with a 90‐s enclosure period and a 30‐s delay between measurements. The final instantaneous *R*_s_ for a given plot consisted of the average of the three measurements, and if necessary, one or more additional measurements were taken until the coefficient of flux variation was below than 2%. The measurements were conducted from August to October in 2009 and May to October in 2010. When measuring *R*_s_, *T*_s_ was determined at a depth of 5 cm adjacent to each PVC collar with a thermocouple. Simultaneously, *S*_w_ at a depth of 5 cm was measured using a Theta Probe Soil Moisture Sensor. Field meteorological data were obtained from the local meteorological station.

### 2.3 Biomass and soil characteristics

AGB and BGB were collected and measured at the end of every month (May to September) in 2010, five representative 1 × 1 m quadrats were established at each site. For determination of AGB, all plants from five quadrats were clipped above the soil surface at each site, and litter was collected using rake. After removal of AGB and litter biomass, five belowground core samples at the depth of 0–30 cm were collected from each quadrant using a soil auger with a diameter of 8 cm. The root was separated from the soil by washing over a 0.2‐mm mesh to determine BGB. All plant samples were dried at 65°C to constant weight for biomass determination.

On 15 August 2010, three soil samples were collected in each plot using a soil auger with a diameter of 4 cm at a depth of 0–30 cm at 10‐cm intervals, the samples were mixed to obtain one composite sample per plot. Subsequently, the samples were sieved (<2 mm) and any roots were removed. Then, samples were ground in a ball‐bearing mill and sieved (<0.9 mm) prior to analysis of SOC. We determined SOC using the K_2_Cr_2_O_7_–H_2_SO_4_ digestion method (Nelson & Sommers, 1982). Soil bulk density was determined using soil cores (volume of 100 cm^3^) obtained from depths of 0–10 cm.

### 2.4 Data analysis

Soil respiration, *T*_s_, and *S*_w_ were calculated by averaging the five replicates on each sampling day. We conducted all statistical analyses using SPSS 19.0 (SPSS Inc., Chicago, IL, USA). Spatial variation of *R*_s_ in all sites was analyzed using one‐way ANOVA. There was no temporal autocorrelation of soil respiration with soil temperature and water content according to the autocorrelation function (ACF) test; consequently, regression analysis was conducted between *R*_s_ and *T*_s_, *S*_w_. Multiple regression analysis was used to examine the relationships between *R*_s_ and *T*_s_, *S*_w_. Least significant difference (LSD) was used to evaluate differences between sites, and analyses were performed at significance levels of *p *< .05, *p *< .01, and *p *< .001. Monthly cumulative *R*_s_ (gC/m^2^) were calculated by linear interpolation of daily *R*_s_ from 5 August to 15 October in 2009 and from 5 May to 31 October in 2010.

To simulate the relationship between *R*_s_ and *T*_s_, we applied the exponential regression model:

$$R_{s}=\alpha e^{\beta T_{s}}$$

where *R*_s_ is the soil respiration rate (μmol CO_2_ m^−2^ s^−1^), *T*_s_ is the soil temperature (°C) at a depth of 5 cm, and β is constants fitted by the models.

The *Q*_10_ values were calculated with the Van't Hoff model:

$$Q_{10}=e^{\beta \times 10}$$

## 3 RESULTS

### 3.1 Abiotic and biotic factors among four soil management types

**Figure 1:**
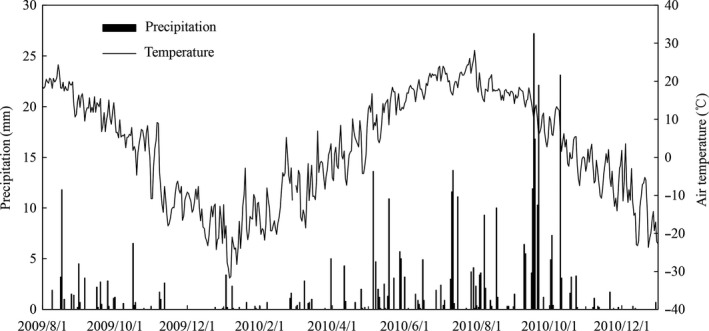
Dynamic of precipitation and air temperature from 1 August 2009 to 31 December 2010

**Figure 2:**
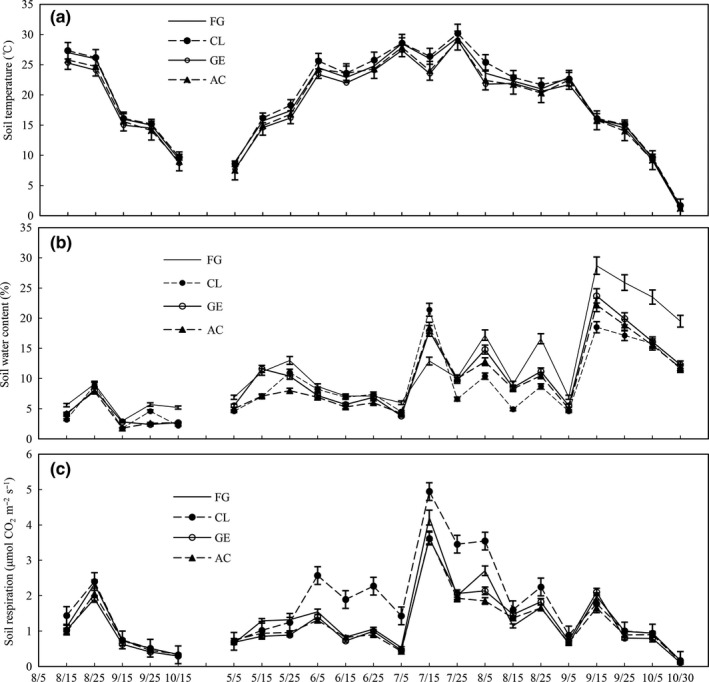
Soil temperature (°C, 0–5 cm), soil water content (%, 0–5 cm), and soil respiration (μmol CO_2_ m^−2^ s^−1^) for free grazing grassland (FG), cropland (CL), grazing enclosure grassland (GE), and abandoned cultivated grassland (AC) in 2009 (August to October) and 2010 (May to October). (a) soil temperature, (b) soil water content, and (c) soil respiration. Bars indicate mean ± standard error

Changes in *T*_s_ coincided with air temperature, and the maximum and minimum *T*_s_ values were observed in July and October (Figures 1 and 2a); levels of *S*_w_ at the depth of 0–5 cm coincided with that of irregular rainfall, with the highest values in September due to high precipitation (Figures 1 and 2b). Levels of *T*_s_ significantly decreased from 21.9 ± 0.3 to 20.7 ± 0.2°C and 21.3 ± 0.1 to 20.4 ± 0.3°C with the conversion of CL to AC and FG to GE, respectively (*p *< .05). *S*_w_ significantly decreased from 13.6 ± 0.5% to 10.2 ± 0.7% and 11.5 ± 0.4% with the conversions of FG to CL and GE (*p *< .05).

The biotic parameters also varied significantly with soil management changes (*p *< .05). In particular, SOC decreased from 33.07 ± 0.05 g/kg to 11.78 ± 0.28 and 17.98 ± 0.87 g/kg with conversions of FG to CL and GE. Similarly, mean BGB during growing season decreased from 1570.7 ± 198.6 g/m^2^ to 532.6 ± 100.0 and 1141.1 ± 122.2 g/m^2^ with the conversions of FG to CL and GE. Furthermore, mean AGB during the growing season significantly increased from 57.70 ± 10.0 to 246.62 ± 20.3 g/m^2^ with the conversion of FG to CL (Table 1).

**Table 1:** Vegetation and soil characteristics for free grazing grassland (FG), cropland (CL), grazing enclosure grassland (GE), and abandoned cultivated grassland (AC) (values represent mean ± standard error, *n* = 5). Different letters in each column indicate significant differences at *p* < .05

Soil management	Bulk density (g/cm^3^, 0–10 cm)	SOC (g/kg, 0–30 cm)	AGB (g/m^2^)	BGB (g/m^2^, 0–30 cm)	Litter biomass (g/m^2^)
FG	1.30 ± 0.0b	33.07 ± 0.05a	57.70 ± 10.0c	1570.7 ± 198.6a	10.11 ± 5.84c
CL	1.21 ± 0.04c	11.78 ± 0.28c	246.62 ± 20.3a	532.6 ± 100.0d	–
GE	1.37 ± 0.03ab	17.98 ± 0.87b	177.78 ± 15.2b	1141.1 ± 122.2b	75.39 ± 9.31a
AC	1.42 ± 0.02a	14.69 ± 1.45b	142.72 ± 13.9b	873.3 ± 116.2c	37.75 ± 4.21b

SOC, soil organic carbon; AGB and BGB, average aboveground and belowground biomass from May to September in 2010.

### 3.2 Dynamic change in soil respiration

**Figure 3:**
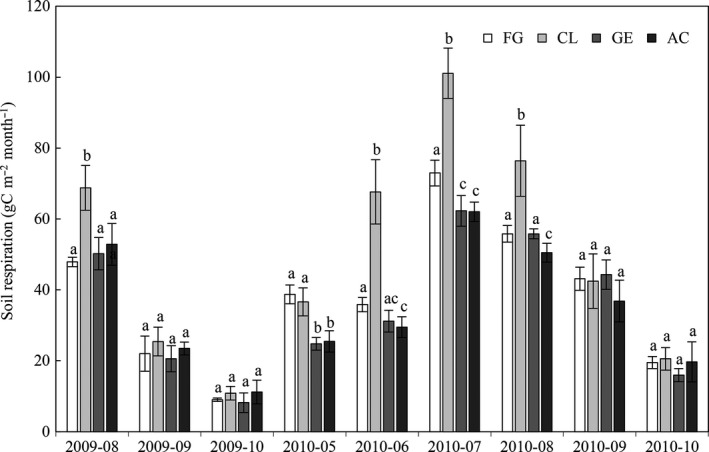
Seasonal dynamic of monthly cumulative respiration (gC m^−2^ month^−1^) for free grazing grassland (FG), cropland (CL), grazing enclosure grassland (GE), and abandoned cultivated grassland (AC) in 2009 (August to October) and 2010 (May to October). Bars indicate mean ± standard error. Different letters indicate significant differences at *p *< .05

The *R*_s_ showed similar daily variations in the four soil management types. The extremely low *R*_s_ values coincided with a drought on July 5, and after the onset of rain on July 11, *R*_s_ sharply increased to its highest values of the growing season on July 15 (Figure 2c). The dynamics of monthly cumulative *R*_s_ coincided with *T*_s_ and air temperature, with maximum values in July and minimum values in October (Figure 3). The cumulative *R*_s_ followed the order of CL (344.74 gC m^−2^ year^−1^) > FG (265.97 gC m^−2^ year^−1^) > GE (236.70 gC m^−2^ year^−1^) > AC (226.42 gC m^−2^ year^−1^) during the growing season in 2010 (Table 2). Furthermore, *R*_s_ in 2010 was about 1.3 to 1.5 times higher than the corresponding values in 2009, possibly due to higher precipitation in 2010 than 2009 (363 vs. 248 mm) (Table 2).

**Table 2:** Daily mean soil respiration (R, μmol CO_2_ m^−2^ s^−1^) and cumulative soil respiration (CR, gC/m^2^) during the growing seasons from 2009 to 2010 for free grazing grassland (FG), cropland (CL), grazing enclosure grassland (GE) and abandoned cultivated grassland (AC) (values represent mean ± standard error, *n* = 5). Different letters in each column indicate significant differences at *p* < .05

Soil management	R	CR		
	2010	2009	2010	
	(May to October)	(August to October)	(May to October)	(August to October)
FG	1.43 ± 0.04b	80.93 ± 1.10b	265.97 ± 7.49b	118.45 ± 9.23b
CL	1.85 ± 0.15a	105.02 ± 9.80a	344.74 ± 28.42a	139.42 ± 16.02a
GE	1.27 ± 0.04c	78.96 ± 8.91ba	236.70 ± 8.33c	116.06 ± 4.64b
AC	1.21 ± 0.06c	84.18 ± 7.60ba	226.42 ± 12.63c	107.02 ± 10.38b

### 3.3 Correlation between *R*_s_ and *T*_s_, *S*_w_

**Figure 4:**
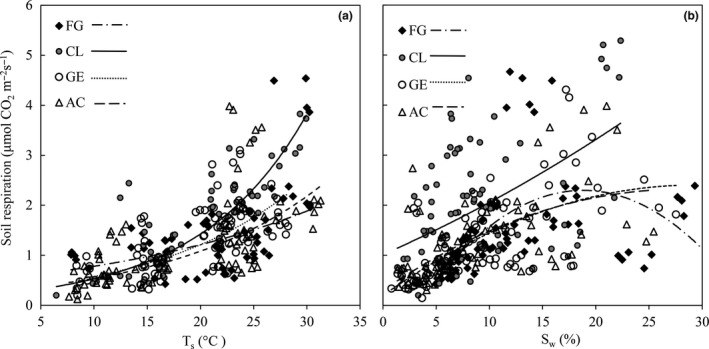
The relationship between daily mean *R*_s_ (μmol CO_2_ m^−2^ s^−1^) and soil temperature (*T*_s_, °C, 0–5 cm), soil water content (*S*_w_, %, 0–5 cm) for free grazing grassland (FG), cropland (CL), grazing enclosure grassland (GE), and abandoned cultivated grassland (AC). (a) relationship between *R*_s_ and *T*_s_; (b) relationship between *R*_s_ and *S*_w_

For the four soil management types, daily mean *R*_s_ significantly increased exponentially with *T*_s_ (*p* < .001). *T*_s_ explained 26%–70% of the variation in *R*_s_ (Figure 4, Table 3). Although the relationship between *R*_s_ and *T*_s_ was similar among the four soil management types, the coefficient of determination (*R* ^2^) was greater in CL than FG and AC (*R* ^2^: 70% vs. 26% and 49%), and lower in FG than that in GE (*R* ^2^: 26% vs. 50%) (Table 3). Values of *Q*_10_ were 1.55, 2.66, 2.10, and 2.01 in FG, CL, GE, and AC, respectively (Table 3).

**Table 3:** Regression equations for daily mean *R*_s_ (μmol CO_2_ m^−2^ s^−1^) with soil temperature (°C, 0–5 cm) and soil water content (%, 0–5 cm) for free grazing grassland (FG), cropland (CL), grazing enclosure grassland (GE), and abandoned cultivated grassland (AC)

Soil management	*R*_s_ = a*e^b*Ts^	*R*_s* *_= a*S_w_^2^ + b*S_w_ + c	*Q*_10_
FG	*R*_s_ * *= 0.501e^0.044 *Ts*^, *R* ^2^ * *= .26, *p *< .001	*R*_s_ * *= −0.009 *S*_w_^2^ + 0.350 *S*_w_ − 1.000, *R* ^2^ = .26, *p *< .001	1.55
CL	*R*_s_ * *= 0.197e^0.098 *Ts*^, *R* ^2^ = .70, *p *< .001	*R*_s_ * *= 0.001 *S*_w_^2^ + 0.093 *S*_w_ + 1.015, *R* ^2^ = .26, *p *< .001	2.66
GE	*R*_s_ * *= 0.272e^0.074 *Ts*^, *R* ^2^ = .50, *p *< .001	*R*_s_ * *= −0.002 *S*_w_^2^ + 0.150 *S*_w_ + 0.195, *R* ^2^ = .37, *p *< .001	2.10
AC	*R*_s_ * *= 0.266e^0.070 *Ts*^, *R* ^2^ = .49, *p *< .001	*R*_s_ * *= −0.002 *S*_w_^2^ + 0.144 *S*_w_ * *+ 0.300, *R* ^2^ = .40, *p *< .001	2.01

**Figure 5:**
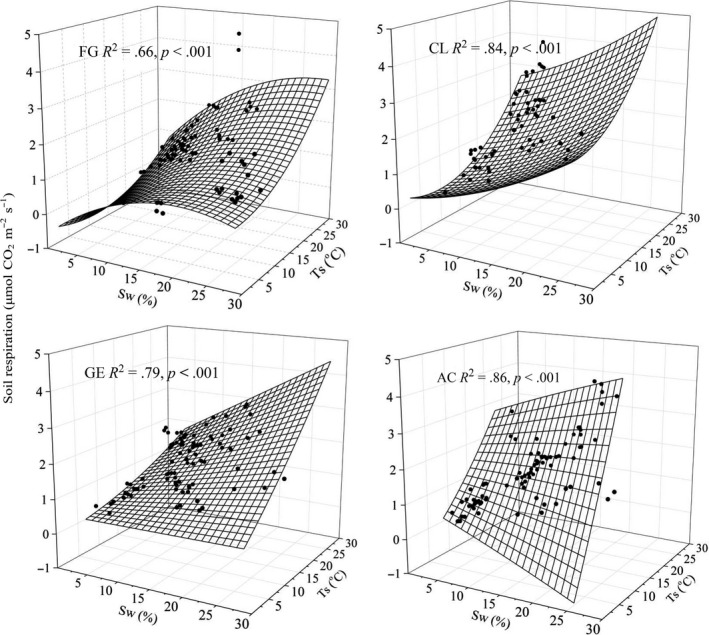
Correlation of daily mean *R*_s_ (μmol CO_2_ m^−2^ s^−1^) with soil temperature (*T*_s_, °C, 0–5 cm), soil water content (*S*_w_, %, 0–5 cm) for free grazing grassland (FG), cropland (CL), grazing enclosure grassland (GE), and abandoned cultivated grassland (AC). The regression equations for each curve are listed in Table 4

**Figure 6:**
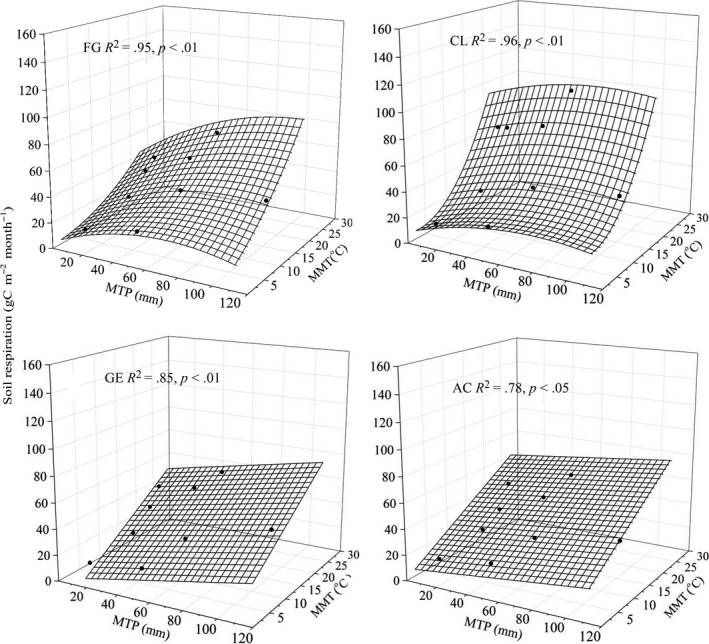
The relationship between monthly cumulative *R*_s_ (gC m^−2^ month^−1^) and monthly total precipitation (MTP), monthly mean temperature (MMT) for free grazing grassland (FG), cropland (CL), grazing enclosure grassland (GE), and abandoned cultivated grassland (AC). The regression equations for each curve are listed in Table 4

Daily mean *R*_s_ was significantly correlated with *S*_w_ (*p* < .001) and followed parabolic pattern for four soil management types. The *S*_w_ explained 26%–40% of the variation in *R*_s_ (Figure 4, Table 3). When *S*_w_ values were above about 2%, *R*_s_ increased with the increasing of *S*_w_ in CL, whereas *R*_s_ will decrease when *S*_w_ reached the threshold for the other sites. Multiple polynomial regression analysis showed that *T*_s_ and *S*_w_ explained 66%–84% of the variation in daily mean *R*_s_ (Figure 5, Table 4), while total monthly precipitation (MTP) and monthly mean temperature (MMT) explained 78%–96% of the variation in monthly cumulative *R*_s_ (Figure 6, Table 4).

**Table 4:** Regression equations for monthly cumulative *R*_s_ (gC m^−2^ month^−1^) and monthly total precipitation (MTP), monthly mean temperature (MMT) for free grazing grassland (FG), cropland (CL), grazing enclosure grassland (GE), and abandoned cultivated grassland (AC)

Soil management	*R*_s_ = a∙*T*_s_^2^ + b∙*S*_w_^2^ + c∙*T*_s_∙*S*_w_ + d∙*T*_s_ + e∙*S*_w_ + f	*R*_s_ * *= a∙*MMT* ^2^ + b∙*MTP* ^2^ + c∙*MMT*∙*MTP *+ d∙*MMT* + e∙*MTP *+ f
FG	*R*_s_ * *= 0.004∙*T*_s_^2^ + 0.003∙*S*_w_^2^ + 0.002∙*T*_s_ *S*_w_ + 0.164∙ *T*_s_ −0.063∙*S*_w_−0.674	*R*_s_ * *= 0.073∙*MMT* ^2^0.004∙*MTP* ^2^ + 0.017∙*MMT*∙*MTP* + 0.489∙*MMT* + 0.043∙*MTP* + 1.806
CL	*R*_s_ * *= 0.002∙*T*_s_^2^ + 0.005∙*S*_w_^2^ + 0.001∙*T*_s_∙*S*_w_ + 0.017∙ *T*_s_ − 0.073∙*S*_w_ + 0.396	*R*_s_ * *= 0.003∙*MMT* ^2^ + 0.277∙*MTP* ^2^ + 0.001∙*MMT*∙*MTP* + 0.440∙*MMT* − 2.549∙*MTP* + 10.938
GE	*R*_s_ * *= 0.001∙*S*_w_^2^ + 0.004∙ *T*_s_∙*S*_w_ − 0.017∙*T*_s_ + 0.002∙*S*_w_ + 0.202	*R*_s_ * *= 0.240∙ *MMT* + 2.532∙ *MTP* − 9.184
AC	*R*_s_ * *= 0.002∙*T*_s_^2^ + 0.003∙*S*_w_^2^ + 0.008∙ *T*_s_^2^∙*S*_w_ + 0.115∙ *T*_s_ − 0.099∙*S*_w_ − 0.354	*R*_s_ * *= 2.243∙ *MMT* + 0.139∙ *MTP* − 1.791

### 3.4 Soil respiration among soil management changes

Values of the daily mean *R*_s_ during growing season varied with soil management changes, and it significantly increased by 29% with the conversion of FG to CL and decreased by 35% and 11% with the conversion of CL to AC and FG to GE during the growing season in 2010 (Table 2). From June to August, *R*_s_ in CL was significantly higher than that in the other site types (*p *< .001), while at the end of the growing seasons, we observed no significant difference of *R*_s_ between the four soil management types (Figure 4).

## 4 DISCUSSION

### 4.1 Soil respiration under four different soil management types

In this study, daily mean *R*_s_ values of grassland during the growing seasons ranged from 1.21 to 1.43 μmol CO_2_ m^−2^ s^−1^, which were lower than that in the northern grassland in China (1.87 μmol CO_2_ m^−2^ s^−1^) (Zhang et al., 2015). Again, daily mean *R*_s_ during the growing seasons in CL (1.85 μmol CO_2_ m^−2^ s^−1^), was also lower than that in the agricultural ecosystem in the northern China plain (3.95 μmol CO_2_ m^−2^ s^−1^) (Zhang et al., 2013). The lower *R*_s_ observed in our study is possibly due to lower precipitation (382 vs. 532 mm and 560 mm for cropland and grassland, respectively). Cumulative *R*_s_ ranged from 226.42 to 344.74 gC/m^2^ during the growing seasons, and it fell right into the range reported for temperate grassland (range: 132–830 gC/m^2^) (Raich & Schlesinger, 1992), and close to the study in the same region (262–309 gC/m^2^) (Gong et al., 2014).

Different ecosystem types, in regard to different plant community patterns within an ecosystem, exhibit different *Q*_10_ values. In our study, *Q*_10_ values ranged between 1.55 and 2.66 for the four soil management types, which fell right into the range of the *Q*_10_ in China (range: 1.28–4.75) (Zheng et al., 2009). Values of *Q*_10_ values far greater in CL than that in the other sites, which confirmed previous conclusion that cropland may be more sensitive to climate warming, whereas grassland may have adapted to climatic warming (Tungate, Israel, Watson, & Rufty, 2007). In FG, *Q*_10_ was 1.55, which was close to the lowest value of the range, study has indicated that small quantities of fungi and bacteria may result in lower *Q*_10_ values in grazed grasslands in China (Cao et al., 2004).

### 4.2 Effects of abiotic factors on *R*_s_ in different soil management types

Generally, *T*_s_ and *S*_w_ are considered two of the most important abiotic factors controlling temporal variations of *R*_s_ (Raich & Potter, 1995; Rong et al., 2015). In fact, all biogeochemical processes associated with *R*_s_ inevitably relate to *T*_s_ and *S*_w_ (Risch, Haynes, Busse, Filli, & Schuetz, 2013), and the dependence of *R*_s_ on *T*_s_ and *S*_w_ could be explained by the influence of *T*_s_ and *S*_w_ on availability of carbon substrate (Campbell & Law, 2005). Although the relationship between *R*_s_ and *T*_s_ and *S*_w_ is similar, the coefficient of determination (*R* ^2^) was different (Table 3), suggesting that soil management changes changed the *R*_s_ through influencing the relationship between *R*_s_ and abiotic factors.

The effect of *S*_w_ on *R*_s_ is complex, as soil water affects not only enzymatic activities and physiological processes, but also gas diffusion (Balogh et al., 2011; Unger, Maguas, Pereira, David, & Werner, 2010). Low values of *S*_w_ slow down solute diffusion and limit the supply of organic substrate for microorganisms (Moyano, Manzoni, & Chenu, 2013). In our study, *S*_w_ had the threshold in grassland ecosystem, above or below the threshold, *R*_s_ would decrease, but values of *R*_s_ for CL were almost positively related to *S*_w_, and the result agreed with finding of Rong et al. (2015).

Although the result suggested that *T*_s_ was more important than *S*_w_ in determining soil respiration during growing seasons (Table 3), the dramatic change in *S*_w_ has a significant influence on *R*_s_. Conant, Dalla‐Betta, Klopatek, and Klopatek (2004) indicated that *S*_w_ had an overriding influence on *R*_s_, particularly during the dry season in semi‐arid environments. In a similar study, Rey et al. (2011) found that *S*_w_ was the main driver of *R*_s_ for most of the year when soil temperatures were above 20°C in semi‐arid steppe ecosystems of Spain. In our study, dramatic increase or decrease in *R*_s_ were observed after rainfall or dry events for all soil management types (Figure 2), and the result was in agreement with findings from the other studies (Rey et al.,^2011^; Rong et al., 2015), suggesting that the dramatic change in *S*_w_ have a pronounced influence on *R*_s_.

Abiotic factors explained less daily variation of *R*_s_ in FG than that in the other sites (*R* ^2^: 66% vs. 79%–86%, Figure 5), and it indicated that other factors also play an important role in *R*_s_ in FG. Grazing significantly changes soil physical and chemical characteristics, including SOC, soil bulk density, and soil texture (Bremer, Ham, Owensby, & Knapp, 1998; Gong et al., 2014; Wilsey, Parent, Roulet, Moore, & Potvin, 2002), so the terms above should be considered to improve the explanation of daily mean *R*_s_ in FG.

### 4.3 Effects of soil management changes on soil respiration

Soil management changes result in changes in the soil microclimate and the biotic factors such as vegetation structure, primary productivity, and soil organic matter, thus indirectly affecting *R*_s_ (Chen et al., 2006; Gong et al., 2014; Rong et al., 2015). Values of *R*_s_ increased by 29% due to the conversion of FG to CL, and the result is similar to previous studies that conversion of natural grassland to cropland can increase *R*_s_ (Frank et al., 2006; Wang, Liu, et al., 2015). Changes in soil microclimate due to soil management changes could not explain the increased *R*_s_. *S*_w_ decreased, while there was no significant change in *T*_s_, whereas *R*_s_ increased with the conversion of FG to CL. This significant change may be explained as follows: First, *Q*_10_ was higher for CL compared to FG, which suggests that soil management change from FG to CL could increase the temperature sensitivity of soil respiration and lead to a concomitant loss of soil carbon storage. Second, soil management change from FG to CL increased *R*_s_ through decreasing soil carbon levels (Xie et al., 2007) and soil bulk density. In this study, SOC and soil bulk density in cropland were both significantly lower than that in the FG (Table 1), suggesting that annual tillage enhanced substrate availability and soil aeration, which in turn may have led to increased soil microbial activity and decomposition of soil organic carbon, resulting in a rapid increase in *R*_s_. Third, the higher *R*_s_ in CL may relate to higher AGB, plant photosynthesis has a driving effect on *R*_s_ (Tang, Baldocchi, & Xu, 2005), and *R*_s_ increase with increase in AGB (Gong et al., 2014). In this study, AGB was significantly higher in CL than in FG and AC (Table 1), indicating that the higher AGB promote the release of CO_2_ in CL. Furthermore, manure application also affects the *R*_s_ process in CL. A study in a Mediterranean maize (*Zea mays* L.)‐based cropping system assessed the effect of different fertilization regimes on *R*_s_, with the result that manure fertilization increased *R*_s_ (Lai et al., 2017). In our study, manure fertilization of cattle slurry was used in CL site, which may promote the emission of soil CO_2_.

Soil management change from CL to AC remarkably decreased *R*_s_ by 35%, and it may relate to lower *T*_s_, *Q*_10_ and higher soil bulk density in AC. When the cropland converted to abandoned cultivated grassland, SOC increased and AGB decreased (Table 1), which was conducive to the accumulation of soil organic matter and decrease the release of CO_2_. The result differed from the finding of Wang, Liu, et al. (2015), which showed that *R*_s_ was increased with the conversion of CL to AC. This may be due to shorter restoration year (10 vs. 15 years), which result in lower accumulation of litter biomass (38 vs. 103 g/m^2^). Litter is the main source of soil organic carbon and provides substrate for soil microbial activity, resulting in heterotrophic respiration (Ngao, Epron, Brechet, & Granier, 2005), and it is one of the main factors affecting *R*_s_ along the restoration chronosequence (Wang, Liu, et al., 2015). Our results suggest that after a proper natural restoration period, restoration grasslands should be utilized properly to decrease *R*_s_.

The values of *R*_s_ significantly decreased by 11% with the conversion of FG to GE. On the one hand, *T*_s_ and *S*_w_ both were significantly decreased with soil management change from FG to GE (Figure 2a, b), which lead to the decrease in *R*_s_. On the other hand, grazing animals deposit large amounts of manure that could increase SOC and BGB in FG (Table 1) and, consequently, increased *R*_s_. The active carbon pool in the soil directly provides respiration substrate for decomposition and *R*_s_ increase with the increase of SOC (Smith 2003; Chen, Huang, & Zou, 2010), while root respiration accounts for 13%–55% of total *R*_s_ in temperate grasslands (Gong et al., 2014). The conversion of FG‐ to GE‐induced changes in soil microclimate also contributed to the relatively high *Q*_10_ values (Table 3); the same result also has been reported for the Yellowstone National Park and Tibetan Plateau (Chen et al., 2016; Chuckran & Frank, 2013), and it implies that the carbon stored in the soils of the GE may be particularly vulnerable to the climate warming. Moreover, litter biomass was significantly higher in GE than that in FG (Table 1), and it will continuously increase with restoration years. Given these results, we predict that the rate of CO_2_ release is faster in GE than that in FG if the climate warming.

## 5 CONCLUSIONS

Our findings support the hypotheses that most of the daily and seasonal variation in soil respiration could be explained by soil temperature and soil water content, and soil respiration is significantly affected by soil management changes. This study monitored the effects of soil management changes from free grazing grassland to cropland and grazing enclosure grassland, cropland to abandoned cultivated grassland on soil respiration in Inner Mongolia, China, and it is critical for enhancing our understanding of the global carbon cycle and has practical implications for grassland management. Soil temperature and soil water content significantly influenced soil respiration for all soil management types and explained 66%–86% of the variability in daily soil respiration. Monthly mean temperature and precipitation explained 78%–96% of the variability in monthly cumulative soil respiration. The results showed that soil respiration increased by 29% with the conversion of free grazing grassland to cropland and decreased by 35% and 11% with the conversion of cropland to abandoned cultivated grassland and free grazing grassland to grazing enclosure grassland. The increase in *Q*_10_ and litter biomass induced by the conversion of free grazing grassland to grazing enclosure grassland could lead to increased CO_2_ emissions if the climate warming.

Given the limitations of biotic factors data of this study, soil organic carbon and bulk density were only determined once a year, and biomass was only measured once a month from May to September in 2010, further studies are worthwhile to evaluate the influence of biotic factors (soil organic carbon, aboveground biomass, root biomass, and litter biomass) on soil respiration, and to detect relative contribution of different factors to soil respiration in different soil management types. Furthermore, the studies about the contribution ratio of root and heterotrophic respiration to soil respiration under soil management changes are also needed to fully explain the effect of soil management changes on soil respiration.

## CONFLICT OF INTEREST

None declared.

## AUTHOR CONTRIBUTIONS

Haili Xue analyzed the data and wrote the paper. Haiping Tang designed the research and revised the manuscript. All authors have read and approved the final manuscript.
